# Effects of Smart Position Only (SPOT) Tag Deployment on White Sharks *Carcharodon carcharias* in South Africa

**DOI:** 10.1371/journal.pone.0027242

**Published:** 2011-11-14

**Authors:** Oliver J. D. Jewell, Michelle A. Wcisel, Enrico Gennari, Alison V. Towner, Marthán N. Bester, Ryan L. Johnson, Sarika Singh

**Affiliations:** 1 Mammal Research Institute, University of Pretoria, Hatfield, Pretoria, South Africa; 2 Dyer Island Conservation Trust, Kleinbaai, Western Cape, South Africa; 3 Research, Oceans Research, Mossel Bay, Western Cape, South Africa; 4 ADU Zoology Department, University of Cape Town, Ronderbosch, Western Cape, South Africa; 5 South African Institute for Aquatic Biodiversity, Grahamstown, South Africa; 6 Department of Environmental Affairs, Cape Town, Western Cape, South Africa; Smithsonian's National Zoological Park, United States of America

## Abstract

We present 15 individual cases of sub-adult white sharks that were SPOT tagged in South Africa from 2003–2004 and have been re-sighted as recently as 2011. Our observations suggest SPOT tags can cause permanent cosmetic and structural damage to white shark dorsal fins depending on the duration of tag attachment. SPOT tags that detached within 12–24 months did not cause long term damage to the dorsal fin other than pigmentation scarring. Within 12 months of deployment, tag fouling can occur. After 24 months of deployment permanent damage to the dorsal fin occurred. A shark survived this prolonged attachment and there seems little compromise on the animal's long term survival and resultant body growth. This is the first investigation detailing the long term effects of SPOT deployment on the dorsal fin of white sharks.

## Introduction

Monitoring the large scale movements of pelagic animals is logistically difficult due to the vast spatial ranges they transverse. Transmissions from satellite tags can not penetrate the water's surface, and acoustic telemetry requires receivers to be within a limited range to pick up tag transmission. The last decade of pelagic marine animal research has shown that satellite telemetry has greatly enhanced the documentation of these movements [1]–[5]. For white sharks (*Carcharodon carcharias*) specifically, two types of satellite tag have been used: Pop-off Archival Tags (PAT) and Smart Position Only/Temperature transmitting Tags (SPOT) [6]–[10]; PAT tags are considered to be low stress generating and a relatively non-invasive method of satellite tagging. White sharks are lured close to a research vessel and the tag is attached below the dorsal fin using a tagging pole as the free-swimming animal passes the vessel [5], [11]. PAT tags remain attached for a predetermined period (days/months) before automatically “popping-off”, floating to the surface and transmitting a summary of data collected via satellite. In order to access the full archival record of the tag the tag needs to be retrieved. Tracks are determined from the ARGOS positioning system using data collected on light levels, which can then compare sunrise and sunset and estimated location. These tracks may have root mean square errors of 0.89- of longitude and 1.47- of latitude [12]. SPOT tags are manually attached by drilling through the dorsal fin, which requires the shark to be lifted from the water so that it may be operated on. SPOT tags use GPS based satellite telemetry and transmit data whenever the dorsal fin breaks the surface of the water. These are capable of operating for several years and generally have positioning errors under 1 km [3].

The methods used to attach SPOT tags have come under scrutiny from the press, public and conservation societies in the wake of documentaries detailing their deployment on large adult white sharks. Unlike smaller species, white sharks are not easily brought on board a vessel and released back to the ocean unharmed because of their size, weight, and strength. SPOT tags have also been attached to many other species, such as small cetaceans, and similar concerns have been raised about catching methods and long term damage. Tissue degradation and possible infection have been documented in bottlenose dolphin *(Tursiops truncatus)* [13], and tissue degradation of shark fins was suspected but only revealed recently [14]. We examine the long term effects of SPOT tag satellite transmitters placed on the dorsal fins of white sharks in South Africa using long term non-invasive dorsal fin identification of individual white sharks.

## Methods

All data on re-sighted sharks is taken from incidental observations on either commercial cage diving or chumming research vessels. Initial data from SPOT tagging was collected by Marine and Coastal Management and published under Bonfil et al. [5]. We use photos from their archives but took no part in the actual tagging. As a result no ethics committee approval was required.

**Figure 1:**
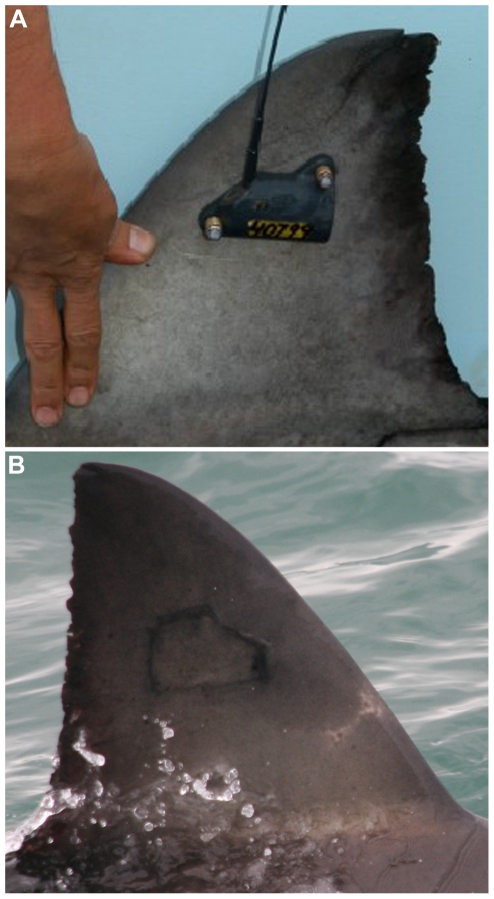
An example of a white shark with SPOT tag freshly deployed (A) and another with pigmentation scaring following SPOT detachment (B).

Archived data from the satellite tagging program [5] was compared to incidental observations from current and archived research and commercial operations. SPOT tags were deployed on 15 white sharks in South African waters between 24^th^ May 2003 and 28^th^ May 2004. These sharks were caught using a double hook baited line from an anchored research vessel. They were then brought on board a purpose built cradle attached to the research vessel and then lifted from the water. Total length (TL) was measured using a straight line to the nearest cm, and tags were then attached to the first dorsal fin using nylon pins, brass washers and steel nuts [5]. Anti-fouling paint was painted on the tag itself and the bolts, but not on the saltwater switch or the antennae, as this was deemed to interfere with transmissions. Tag attachments were designed to keep tags in place for a period of 9–12 months. Digital images of the sharks' dorsal fins were taken before and after deployment of the tags either while in the cradle, as the shark was being hooked, or as the shark was released as in Johnson ([15], Figure 1A) and shark re-sightings were confirmed by matching photographic dorsal fin IDs as in Chapple et al. [16]. Re-sight images were taken either during research operations in Mossel Bay (2005–2011) or on commercial operations aboard the cage diving vessels of Marine Dynamics in Gansbaai (2007–2011).

## Results

**Figure 2:**
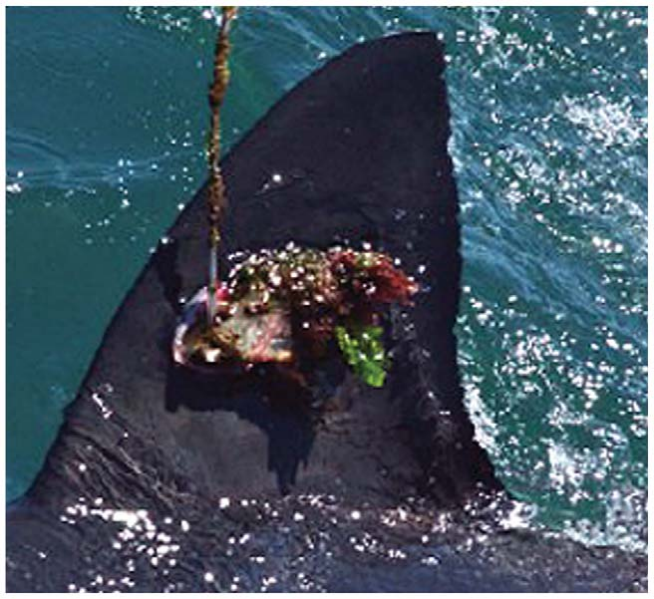
White shark dorsal fin with SPOT tag in place 172 days after deployment with algal growth on tag. Sighted in Mossel Bay November 2003, after making a migration from Mossel Bay to Mozambique and back again.

**Figure 3:**
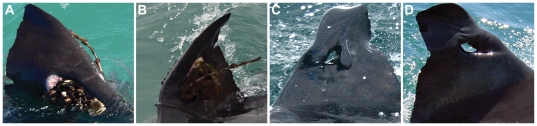
Great white shark dorsal fin with SPOT tag present over 24 months after deployment. (A and B) - tag is showing excessive fouling and fin is leaning to the left as a result of the weight; images taken in 2005 at Mossel Bay and without the tag (C and D) and with resulting hole and fin degradation after tag detachment; images taken in 2009 at Gansbaai.

Eight of the instrumented white sharks were re-sighted without their SPOT tags and with the screw holes healed (Table 1; Figure 1B). One shark (GWS-7) was re-sighted 263 days after deployment, without the tag present but with raw scaring from tag bolts. Two further sharks were re-sighted with SPOT tags still in place. One of these sharks (GWS-1) was re-sighted in Mossel Bay 172 days after tagging. The tag displays fouling, but the shark has not been re-sighted since (Figure 2). The second (GWS-3) was seen in Mossel Bay on 31^st^ August 2005, 822 days after deployment. The tag shows excessive fouling and the shark showed fin deformation with the fin leaning to the left (Figure 3A,B). The tag detached between that sighting in 2005 and subsequent sightings from 2008 onwards. Permanent deformation and a hole remain, leaving the shark extremely distinctive (Figure 3C,D). The shark has been sighted in 2008, 2009, 2010 and 2011 in Gansbaai, and in 2009 and 2010 in Mossel Bay. Observations of the shark's movement indicate that it is relatively unimpeded by the damages to the dorsal fin (Figure S1). The shark also has further unrelated damage to the left pectoral fin which occurred after the original tagging in 2003.

**Table 1:** 

GWS	Size	Date of Tagging	Date of first re-sighting	Days at liberty	Location of sighting	State of dorsal fin/tag
1	280	24/05/2003	12/11/2003	172	Mossel Bay	Tag still present, fouling growth on tag
2	300	01/06/2003	21/06/2004	386	Mossel Bay	Fin healed pigmentation scaring still present
3	290	01/06/2003	31/08/2005	822	Mossel Bay/Gansbaai	Fin degraded and leaning to the left
4	315	07/11/2003	27/03/2010	2192	Gansbaai	Fin healed pigmentation scaring still present
5	330	08/11/2003	25/06/1905	n/a	n/a	Tag deployed - no re-sighting
6	330	15/05/2004	09/03/2005	298	Mossel Bay	Fin healed pigmentation scaring still present
7	300	15/05/2004	02/02/2005	263	Mossel Bay	Tag not present bolt holes still raw
8	300	17/05/2004	23/08/2005	463	Mossel Bay	Fin healed pigmentation scaring still present
9	387	18/05/2004	n/a	n/a	Mossel Bay	Tag deployed - no re-sighting
10	305	18/05/2004	n/a	n/a	Mossel Bay	Fin healed pigmentation scaring still present
11	250	18/05/2004	26/06/2005	404	Mossel Bay	Fin healed pigmentation scaring still present
12	340	20/05/2004	02/05/2005	347	Mossel Bay	Fin healed pigmentation scaring still present
13	391	26/05/2004	n/a	n/a	n/a	Tag deployed - no re-sighting
14	391	26/05/2004	n/a	n/a	n/a	Tag deployed - no re-sighting
15	326	28/05/2004	15/05/2007	1082	Gansbaai	Fin healed pigmentation scaring still present

The size of GWS-3 was estimated at 290 cm when sighted in 2005. Sightings in December 2010 estimated the sharks' length at 375 to 400 cm, using a 400 cm cage for perspective. This suggests a growth of between 85 and 110 cm in 5 years.

**Figure 4:**
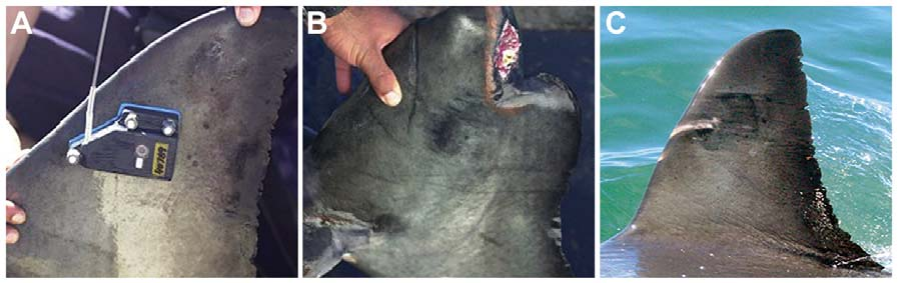
Photo ID of a male white shark tagged in Mossel Bay 2004 with missing upper caudal fin (A and B) re-sighted in Gansbaai 2010 and 2011 from Marine Dynamics cage diving vessel displaying pigmentation scaring from tag (C).

Another shark (GWS-15) tagged on 28/05/2004 was first re-sighted on 27^th^ March 2010 in Gansbaai and was acoustically tagged in 2011. It is identifiable by dorsal fin pigmentation scaring and an amputated upper caudal fin (Figure 4). The caudal fin was healed upon capture (Figure 4B) and as a result is considered an unrelated injury to the tagging project. This shark represents the second oldest re-sight since the original tagging campaign in 2003, and suggests that pigmentation scarring caused by SPOT tags is permanent. The shark is estimated at between 380 and 400 cm TL (based on comparison to cage diameters and taking into account the missing section of the upper caudal). At time of tagging, the sharks' total length was measured at 315 cm, representing an estimated growth of 65–85 cm in TL over 7 years.

## Discussion

The use of SPOT tags in 2003 provided a unique insight into the large scale movement patterns of South African white sharks. However, the use of hooks to catch sharks and drills to attach the tags to the dorsal fins attracted negative press. The use of SPOT tags on white sharks in North America brought further public criticism, particularly in regard to the catching methods. We therefore embarked on the present investigation which represents the first South African record detailing the effects of SPOT tags to white shark dorsal fins after deployment.

Of the original 15 sharks, 8 sharks were sighted with healed fins and pigmentation scars, one with fresh scars suggesting recent tag detachment, one with the tag still in place and one shark with a deformed fin resulting from SPOT tag deployment. These observations suggest that SPOT tags designed to rust and fall out within 12 months are unlikely to cause permanent damage to the structure of the shark's fin as long as they detach within that time. We only observed permanent degradation to the structure of the fin on a shark's tag that was still present for between 24 and 60 months. The shark would have been considered sub-adult at the time of tagging and as a result growth rates would be expected to be relatively quick [17], [18]. Damage to the fins structure was evident from the observation after 24 months. Potential causes of the damage could have been from the impediment of the growth of the fin, pulling it to the left, weight from algal build up on the tag itself which appeared quite excessive 24 months after tag deployment, or quite likely a combination of the two. This result suggests that white sharks yet to obtain full size - particularly while sub-adult and growing fast are unable to sustain SPOT tags in place much longer than 12 months without such damage occurring. Despite the dorsal fin damage to this shark, the shark survived to 2011 and had continued to grow post tag deployment. White sharks can recover from deep tissue wounds (that penetrate skin and muscle) providing vital organs and skeletal structure remain intact [19], however recovery from fin damage is still under investigation and it appears from our results full recovery to the fin has not occurred.

We therefore conclude that based on the tags deployed in South Africa in 2003–2004, SPOT tags did not cause long term damage to the sharks when detached within 12–24 months, but they had the ability to cause permanent structural damage to the dorsal fin when left in place for longer periods. These tags were deployed to relatively sub-adult members of the population (<450 cm) and as such it may be recommended that a review of the tag design is considered for long term deployments to sharks of this size. Observing re-sightings of SPOT tagged white sharks in areas such as Guadalupe or South California would allow a comparison to see if adult white sharks are affected in a similar way. The structural damage to the dorsal fin caused by SPOT tags did not appear to negatively effect the long term survival of the shark and the re-sighting of individuals post tagging and of tracks of individuals not re-sighted suggest there were no mortalities as a result of this programme. However, the effects of removing large (>450 cm) white sharks from the water in order to deploy SPOT tags are still unknown and should also be considered.

## Supporting Information

Figure S1 **White shark displaying damage to the dorsal fin as a result of SPOT tag deployment breaks the water at Gansbaai, South Africa during a Marine Dynamics cage diving trip.** Photo courtesy of Michelle Wcisel, Marine Dynamics.
(TIF)
