# Supplementary figures and images for: Effects of Smart Position Only (SPOT) Tag Deployment on White Sharks Carcharodon carcharias in South Africa
Source: PLoS One. 2011 Nov 14;6(11):e27242. doi: 10.1371/journal.pone.0027242 (PMC3215706; doi:10.1371/journal.pone.0027242)

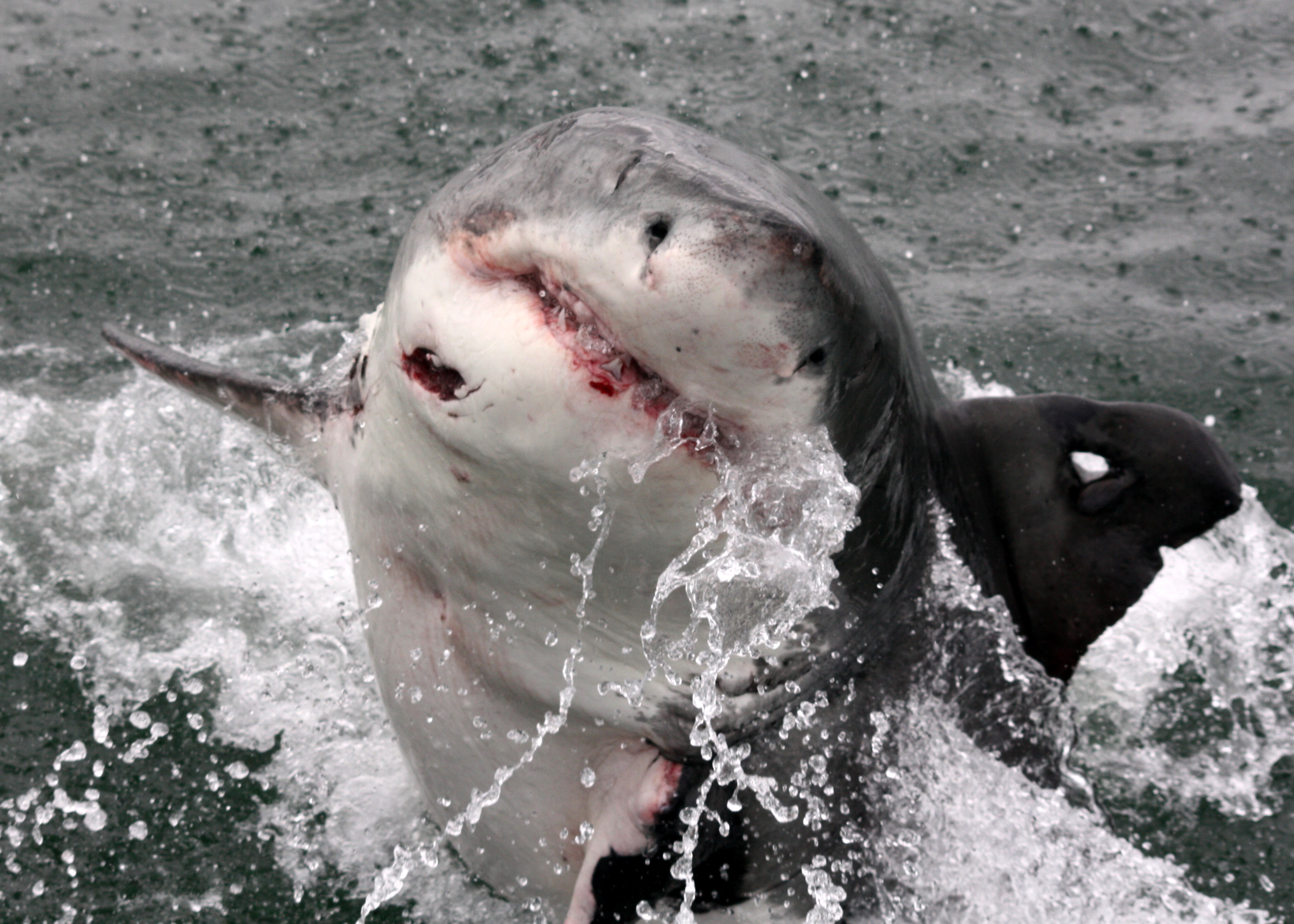

Supplement: Figure S1 — White shark displaying damage to the dorsal fin as a result of SPOT tag deployment breaks the water at Gansbaai, South Africa during a Marine Dynamics cage diving trip. Photo courtesy of Michelle Wcisel, Marine Dynamics. (TIF) [file pone.0027242.s001.tif]
